# Dynamics of Ferroelastic Domain Walls Associated with the Dielectric Relaxation in CsPbCl_3_ Single Crystals

**DOI:** 10.3390/nano16010057

**Published:** 2025-12-31

**Authors:** Zijun Yu, Chen Zou, Dexin Yang

**Affiliations:** 1 College of Materials & Environmental Engineering, Hangzhou Dianzi University, Hangzhou 310018, China; zijunyu@hdu.edu.cn; 2 State Key Laboratory of Extreme Photonics and Instrumentation, College of Optical Science and Engineering, International Research Center for Advanced Photonics, Zhejiang University, Hangzhou 310027, China

**Keywords:** metal halide perovskites, CsPbCl_3_ single crystal, phase transitions, ferroelastic domain walls, dielectric relaxation

## Abstract

Cesium lead chloride (CsPbCl_3_) is a stable, wide-bandgap perovskite with significant potential for ultraviolet (UV) photodetection and blue light-emitting diodes (LEDs). However, the dynamical mechanisms of ferroelastic domain walls associated with the dielectric relaxations in a single-crystal have rarely been reported. In this work, we observed reversible phase transitions from cubic to tetragonal, and further to orthorhombic symmetry, accompanied by the formation and evolution of strip-like ferroelastic domain walls, using in situ X-ray diffraction (XRD), differential scanning calorimetry (DSC), polarized optical microscopy (POM), and dielectric measurements. Notably, the dielectric studies revealed low temperature (~170–180 K) frequency-dependent loss peaks that we attribute to the pinning of polarized domain walls by chloride vacancies. We also found that the formation or disappearance of ferroelastic domain walls near the octahedral tilting transition temperatures leads to pronounced anomalies in the dielectric permittivity. These findings clarify the intrinsic phase behavior of CsPbCl_3_ single crystals and underscore the significant contribution of ferroelastic domain walls to its dielectric response, providing insights for optimizing its optoelectronic performance.

## 1. Introduction

Metal halide perovskites (MHPs) with the *ABX*_3_ structure (where *A* is a monovalent cation such as Cs^+^, methylammonium, or formamidinium; *B* is a divalent metal cation like Pb^2+^, Sn^2+^, or Ge^2+^; and *X* is a halide anion such as Cl^−^, Br^−^, F^−^, and I^−^) have attracted considerable attention for optoelectronic applications [1,2,3,4]. These semiconductors exhibit outstanding optoelectronic properties and have been successfully employed in light-emitting diodes [5,6,7,8,9], solar cells [10,11], and photodetectors [12,13]. In particular, the all-inorganic perovskite CsPbCl_3_ has a wide band gap (2.90 eV) and has been extensively studied for UV-sensitive photodetection [14,15,16,17,18]. Compared to organic-inorganic hybrid perovskites, CsPbCl_3_ exhibits superior thermal and chemical stability [19,20,21,22]. In addition, previous reports using PL and IR spectroscopy have demonstrated that CsPbCl_3_ is a model material system characterized by structural flexibility and tunability, strong excitonic luminescence, and remarkable quantum effects [23,24,25]. Furthermore, its optoelectronic performance can be strongly influenced by factors such as doping, octahedral tilting phase transitions, and microstructures [26,27,28,29,30,31,32,33,34]. These influences offer diverse pathways for engineering the material’s properties and device performance.

CsPbCl_3_ undergoes multiple structural phase transitions within a narrow temperature range (~10 K), but the exact sequence and temperatures reported in the literature vary. Some studies indicate CsPbCl_3_ undergoes octahedral tilting transitions upon cooling, following the sequence *Pm*$\overset{-}{3}$*m* → *P*4/*mbm* → *Cmcm* → *P*2_1_/*m* (or *Pnma*) with transition temperatures of ~320 K, ~315 K, and ~310 K [35,36,37,38]. He et al. reported transitions at 325 K and 316 K, corresponding to the sequence *Pm*$\overset{-}{3}$*m* → *P*4/*mbm* → *Pnma* upon cooling [39]. Meanwhile, there are works suggested a transition pathway of *Pm*$\overset{-}{3}$*m* → *P*4/*mbm* → *Pnma* occurring at 320 K and 310 K upon cooling [40]. Ferroelastic domain walls form when these semiconductors experience a cubic-tetragonal transition during cooling [26]. During subsequent temperature or pressure variations in MHPs, the density, thickness, moment, and mobility of these walls can change dramatically, which may enhance the semiconductors’ optoelectronic properties [26,28]. Therefore, investigating ferroelastic domain walls during these phase transitions is critical for understanding their intrinsic properties and developing CsPbCl_3_-based high-performance optoelectronic devices [41,42,43,44,45].

In this study, we used inverse temperature crystallization (ITC) to grow large bulk CsPbCl_3_ single crystals [46,47,48]. The final synthesis temperature was ~358 K, above the cubic-tetragonal transition. We characterized the crystal structures and composition by room-temperature powder XRD and SEM-EDS. Temperature-dependent XRD and DSC were used to determine the phase transition sequences. The evolution of ferroelastic domain walls was directly observed by in situ polarized optical microscopy (POM) during heating and cooling between ~305 K and 322 K. Finally, we measured the dielectric permittivity and loss (10–350 K) to assess the influence of phase transitions and microstructure (i.e., domain walls) dynamics on the macroscopic dielectric properties.

## 2. Materials and Methods

### 2.1. Single Crystal Growth

CsPbCl_3_ single crystals were synthesized using inverse temperature crystallization (ITC). To grow CsPbCl_3_ single crystals, CsCl powders (>99.99%, Aladdin, Shanghai, China) and PbCl_2_ powders (>99.99%, Aladdin) in a molar ratio of 1:1.4 were dissolved in dimethyl sulfoxide (DMSO, 99.7% with molecular sieves, Water ≤ 50 ppm, Macklin, Shanghai, China) solution with a concentration of 0.07 mol L^−1^. First, the solution was stirred at 373 K for 2 h to ensure complete dissolution of the precursors and to obtain a clear solution. Subsequently, this clarified solution was transferred into an uncapped vial that had been pre-heated to 373 K. The vial was then placed in an oven maintained at 373 K to allow for solvent evaporation until small, regular CsPbCl_3_ single crystal seeds precipitated at the bottom of the vial. The hot saturated perovskite precursor solution was then filtered through a 0.45 μm PTFE filter to remove poor-quality CsPbCl_3_ single crystals, and then the clear solution was transferred to a new vial, and a perforated cap was used to promote solvent evaporation. Afterward, the vial with the perforated cap was placed in silicone oil at 358 K. After being maintained for 2 h, a small CsPbCl_3_ single crystal seed was added to the hot solution and maintained at 358 K. Through continued solvent evaporation over ~3–4 days, the crystals were allowed to grow slowly, yielding the final CsPbCl_3_ single crystal with a size of a few millimeters.

### 2.2. X-Ray Diffraction (XRD)

A CsPbCl_3_ single crystal was ground for room-temperature powder XRD on a SmartLab 9 kW (Rigaku, Tokyo, Japan) diffractometer with Cu-K_α_ radiation (λ = 1.5406 Å) operated at 40 kV and 100 mA. Data collection was performed in continuous scanning mode over a 2θ angular range of 12° to 55°, with a step size of 0.02° and scanning rate of 5° min^−1^. To obtain crystal information, Rietveld refinements were performed with the software TOPAS-Academic V7 [49]. A shifted Chebyshev function with eight parameters, and a pseudo-Voigt function (TCHZ type) were used to fit the background and peak shape, respectively.

In situ heating XRD patterns were obtained on a Rigaku SmartLab diffractometer with Cu-K_α_ radiation source operated at 45 kV and 200 mA. The CsPbCl_3_ powders were measured in a vacuum high-temperature chamber from 103 to 330 K upon heating. The powders were maintained at each temperature for 10 min before measuring. Each XRD spectra were collected from 12° to 55° (2θ) with a step size of 0.02° and scanning rate of 2° min^−1^.

### 2.3. Scanning Electron Microscopy (SEM)

The chemical composition and surface morphology of the as-grown CsPbCl_3_ single crystal were checked using a scanning electron microscope (SEM, JSM-IT500HR/LA, JEOL, Tokyo, Japan) equipped with energy-dispersive X-ray spectroscopy (EDS) at a 15 kV accelerating voltage. The sample was mounted on the specimen stub using conductive adhesive.

### 2.4. Differential Scanning Calorimetry (DSC)

DSC measurement of CsPbCl_3_ single crystals was carried out on a Discovery DSC 25 analyzer (TA Instruments, New Castle, DE, USA) during heating and then cooling between 215 and 372 K at a ramp rate of 10 K min^−1^ under nitrogen atmosphere with a gas flow rate of 50 mL min^−1^.

### 2.5. Polarized Optical Microscopy (POM)

Ferroelastic domain walls were observed by in situ polarized optical microscopy. A (001)-oriented CsPbCl_3_ crystal facet was placed on a temperature-controlled microscope stage (MSD-S690, Murzider, Guangdong, China) under a polarizing optical microscope. The crystal was sandwiched between a glass slide (~0.17 mm thick) and the stage, and was heated in air from room temperature up to 322 K, then cooled back to ~306 K. At key temperatures (~322 K, 320 K, 305 K corresponding to *Pm*$\overset{-}{3}$*m*, *P*4/*mbm*, *Pnma* phases), the sample was held for 10 min to equilibrate before capturing polarized-light micrographs. The same region of the crystal was imaged throughout heating and cooling to accurately track domain wall evolution.

### 2.6. Dielectric Measurements

The dielectric properties of a CsPbCl_3_ single crystal were measured using a Quantum Design PPMS-9T system with a Keysight E4980AL impedance analyzer (Keysight Technologies, Santa Rosa, CA, USA). The single crystal sample used for the measurements had a thickness of ~1.23 mm. A parallel-plate capacitor configuration was prepared by applying silver conductive paint (SPI Supplies, West Chester, PA, USA) to the (001) and opposite faces of the crystal. Each electrode face had dimensions of ~1.50 mm × ~1.50 mm. Thin gold wires (0.5 mm diameter) were used to connect the painted electrodes to the PPMS sample puck using silver paint. After evacuating the sample chamber to <8 Torr, the dielectric permittivity (*ε*) and loss tangent (tan *δ*) were recorded under an AC voltage of 2 V at frequencies from 50 to 300 kHz. Temperature was swept from 10 K to 350 K at 4 K/min under continuous heating and cooling.

## 3. Results and Discussion

### 3.1. Structural and Compositional Characterization

**Figure 1:**
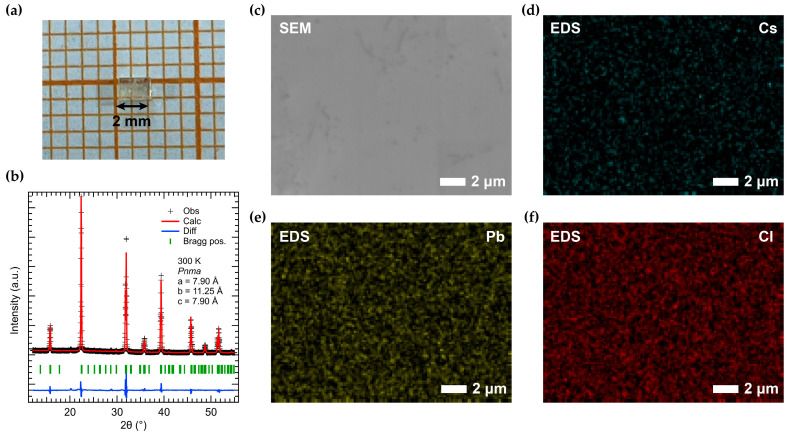
(**a**) Photo of a CsPbCl_3_ bulk single crystal grown by inverse temperature crystallization (ITC). (**b**) Rietveld refinement of room-temperature X-ray diffraction data from CsPbCl_3_ powders for the space group of *Pnma*. The powders were ground from CsPbCl_3_ bulk single crystal. Observed profile is marked by black crosses and calculated profiles are represented by red lines. Differences (experimental minus calculated) are shown in blue. Bragg peak positions are indicated by green marks. (**c**) The SEM image of the surface of a CsPbCl_3_ single crystal, with the corresponding elemental mapping from SEM-EDS shown in (**d**–**f**). The atomic percentages are as follows: Cs 20.43%, Pb 20.81%, Cl 58.76%.

Figure 1a shows a representative CsPbCl_3_ single crystal grown by ITC. Figure 1b presents the Rietveld refinement of the room-temperature powder XRD pattern in the orthorhombic *Pnma* space group. The fit (red line) matches the observed data (black crosses) very well, indicating phase purity. The refined lattice parameters are a = 7.90 Å, b = 11.25 Å, c = 7.90 Å, and these values agree closely with previously reported data at room temperature [50,51]. SEM and EDS mapping were performed to verify the crystal composition. Figure 1c is an SEM image of the as-grown crystal surface, and Figure 1d–f show the elemental maps for Cs, Pb, and Cl. The maps confirm a uniform distribution of all elements. The measured atomic percentages (Cs 20.43%, Pb 20.81%, Cl 58.76%) correspond essentially to the expected 1:1:3 stoichiometry for CsPbCl_3_.

### 3.2. Structural Phase Transitions

**Figure 2:**
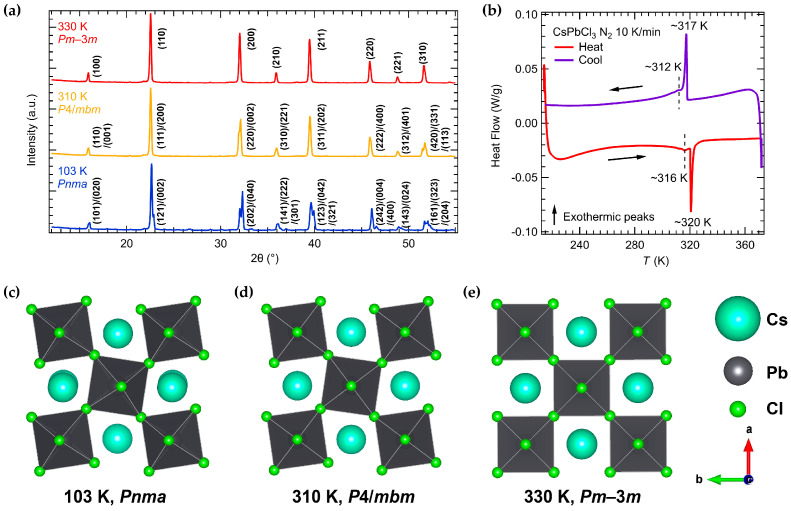
(**a**) XRD patterns of CsPbCl_3_ powders collected at 103, 310, and 330 K. (**b**) Differential Scanning Calorimetry (DSC) curves for a single crystal CsPbCl_3_ measured in nitrogen atmosphere with a scanning rate of 10 K min^−1^. (**c**–**e**) Crystal structures in different group spaces of CsPbCl_3_ at 103 K (**c**), 310 K (**d**), and 330 K (**e**), respectively, from the Rietveld refinement results.

Figure 2a shows selected high-temperature XRD patterns of CsPbCl_3_ powder at 330 K, 310 K, and 103 K. At 330 K, all peaks remain unsplit, consistent with the cubic *Pm*$\overset{-}{3}$*m* phase [52]. On cooling to 310 K, the (200) and (310) peaks begin to split while other peaks like (100) and (110) remain single, indicating a transition to tetragonal *P*4/*mbm* symmetry [53]. At 103 K, further splitting of all peaks is evident, matching the characteristic pattern of orthorhombic *Pnma* phase [54] and the room-temperature XRD pattern in Figure 1b. Figure 2c–e schematically illustrate the CsPbCl_3_ crystal structures in the *Pnma*, *P*4/*mbm*, and *Pm*$\overset{-}{3}$*m* phases, clearly demonstrating the progressive octahedral tilting from cubic through tetragonal to orthorhombic symmetry.

DSC was used to further confirm the structural transitions of a CsPbCl_3_ single crystal between 215 and 372 K. The heat flow (Figure 2b) reveals two thermal anomalies: a larger peak near 320 K (317 K on cooling) and a smaller peak near 316 K (312 K on cooling) [55,56]. The slight differences between the heating and cooling anomalies are likely due to the instrumental thermal lag, as the data were collected under continuous heating/cooling conditions. The sharp anomaly at ~320 K/317 K corresponds to the first order *P*4/*mbm* → *Pm*$\overset{-}{3}$*m* transition [52,55,57], while the weaker anomaly at ~316 K/312 K corresponds to the second order *Pnma* → *P*4/*mbm* transition [12,37,50,57]. These DSC temperatures agree well with the XRD findings (tetragonal phase seen at ~310 K), allowing for a slight offset (2–6 K) due to instrumental and thermal lag. No other transitions were observed down to 103 K.

### 3.3. Evolution of Ferroelastic Domain Walls

**Figure 3:**
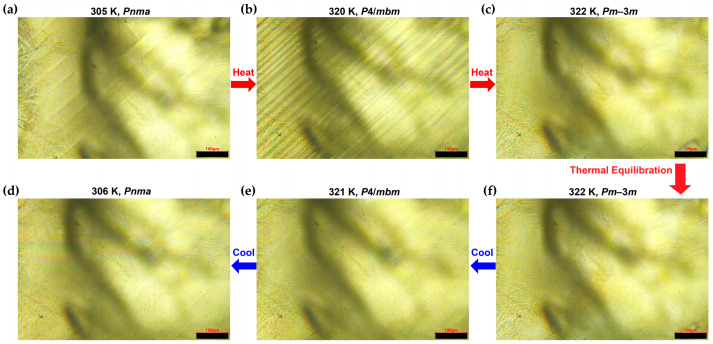
Polarized optical microscopy (POM) of a CsPbCl_3_ bulk single crystal at different temperatures during heating and then cooling process between ~305 K and ~322 K: heating sequence: (**a**) 305 K, (**b**) 320 K, (**c**) 322 K; cooling sequence: (**f**) 322 K, (**e**) 321 K, (**d**) 306 K. The transformations of the ferroelastic domain walls during heating and cooling through orthorhombic-tetragonal-cubic phase transitions are clearly observable. The crystallographic face of the microscopic images is (001). Scale bar: 100 μm.

Ferroelastic domain walls in ferroelastic single crystals form as the crystal symmetry is lowered from cubic to orthorhombic [58,59]. In situ polarized optical microscopy of the (001) face [26,27,28] of a CsPbCl_3_ single crystal, measured during heating from 305 K to 322 K and then cooling from 322 K to 306 K, revealed the dynamics of these domain walls (Figure 3). Figure 3a shows the strip-like ferroelastic domain walls of the as-grown sample at room temperature with the *Pnma* phase. At this point, the domain walls are clear and relatively wide, with each domain measuring ~50–80 μm in width. As the temperature is raised into the tetragonal *P*4/*mbm* phase (e.g., 320 K, Figure 3b), the ferroelastic domain walls begin to move and eventually become densely arranged, with each domain narrowing to only ~10–30 μm in width. Upon further heating into the cubic *Pm*$\overset{-}{3}$*m* phase (322 K, Figure 3c), the ferroelastic domain walls vanish completely.

After holding at the cubic phase for several minutes, the sample was cooled. Upon cooling back into the tetragonal phase (~321 K ± 1 K, near the cubic-tetragonal transition; Figure 3e), the domain walls reappear but are noticeably less dense and less distinct than during heating at ~320 K ± 1 K. At this stage, it is difficult to determine the precise domain width. Lowering the temperature further to the orthorhombic phase (306 K, Figure 3d), broader domains of ~50 μm in width re-formed with a spatial distribution resembling that shown in Figure 3a, although the re-formed walls exhibit lower contrast and appear markedly fainter. Therefore, the evolution of domain walls through the *Pm*$\overset{-}{3}$*m*–*P*4/*mbm*–*Pnma* transitions are partially reversible: they form as dense features in the tetragonal phase and appear as broader stripes in the orthorhombic phase, but they are fully absent in the cubic phase. Throughout the entire heating and cooling cycle, almost all domain walls remain parallel to each other before and after the phase transitions. The reduced density of walls on cooling (as seen in Figure 3d,e) suggests a hysteresis in domain wall formation.

### 3.4. Dielectric Relaxation and Ferroelastic Domain Wall Effects

**Figure 4:**
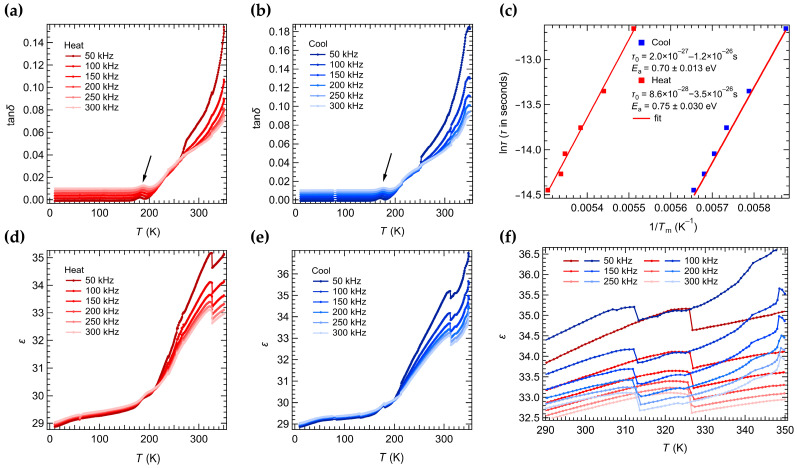
Dielectric loss, tan *δ* (**a**,**b**), and dielectric permittivity, *ε* (**d**,**e**), for a CsPbCl_3_ bulk single crystal measured at frequencies of 50, 100, 150, 200, 250, and 300 kHz during continuous cooling and heating at 4 K min^−1^ between 10 K and 350 K. (**c**) Arrhenius plot from fits to give the temperature, *T*_m_, at which the frequency-dependent peaks in tan *δ* near 180 K had their maximum values. The straight line represents *τ* = *τ*_0_ exp(*E*_a_/R*T*) with *τ*_0_ = 8.6 × 10^−28^–3.5 × 10^−26^ s, *E*_a_ = 0.75 ± 0.030 eV for the heating process and *τ*_0_ = 2.0 × 10^−27^–1.2 × 10^−26^ s, *E*_a_ = 0.70 ± 0.013 eV for the cooling process. (**f**) Enlarged image of the dielectric permittivity curves around ~320 K during heating and cooling.

The dynamics of the ferroelastic domain walls will have a direct influence on the optoelectronic properties in MHPs [26,28]. To assess the influence of ferroelastic domain walls on bulk properties and their dynamical features, we measured the dielectric response of a CsPbCl_3_ crystal (10–350 K) (Figure 4). Figure 4a,b exhibit the frequency-dependent peaks (loss maxima) near ~180 K on heating and ~170 K on cooling, respectively. Plotting the peak temperatures (*T*_m_) in an Arrhenius form (*τ* = *τ*_0_ exp(*E*_a_/R*T*), Figure 4c) gives activation energies of *E*_a_ = ~0.75 ± 0.03 eV (heating) and ~0.70 ± 0.01 eV (cooling) for the relaxation process. These values are comparable to reported domain wall relaxation energies in other materials (e.g., ~0.42–0.65 eV in certain ferrites and multiferroics) [59,60,61,62] and to vacancy-related pinning energies (e.g., ~0.91 V for oxygen vacancies in LaAlO_3_ [63]). In addition, theoretical studies indicate that ferroelastic domain walls can carry a polar response even if the bulk is nonpolar [28,64]. We thus interpret the low-temperature dielectric loss peak as due to the freezing (pinning) of polarized ferroelastic domain walls by chloride vacancies.

Figure 4d,e show the real part of the dielectric permittivity (*ε*) measured during cooling and then heating. We observe sharp anomalies in *ε* at the structural transition temperatures (Figure 4f). Specifically, *ε* exhibits a slight change in slope near 322 K, followed by a sharp drop around 326 K during heating. During cooling, *ε* shows a sharp rise near 312 K and a subtle slope change near 308 K. These dielectric anomalies closely match the transition temperature from DSC and agree with prior reports [57,65]. We attribute the abrupt changes to the first-order cubic–tetragonal transition [52,55,57] and the smaller inflections to the second-order tetragonal–orthorhombic transition [57]. The dielectric transition temperatures differ slightly between heating and cooling, primarily due to thermal lag under continuous scanning of the dielectric measurement.

Importantly, the behavior of *ε* also reflects the dynamics of ferroelastic domain walls [66,67,68,69,70,71]. When the crystal enters the high-symmetry cubic phase on heating, the domain walls disappear, which coincides with the sharp drop in *ε*. Conversely, on cooling, as the crystal exits the cubic phase and ferroelastic domain walls re-form in the tetragonal phase, *ε* rises sharply. The further evolution of domain walls at the lower transition induces the weak slope changes in *ε*. The fainter and less dense domain walls that re-formed upon cooling is correlated with the weaker and less abrupt change in the dielectric permittivity observed during the cooling cycle. In summary, the dielectric permittivity anomalies at the transitions can be largely attributed to the creation or annihilation of polarized ferroelastic domain walls, demonstrating that these walls make an additional contribution to the bulk dielectric response.

## 4. Conclusions

We have grown high-quality bulk CsPbCl_3_ single crystals by a solution-based inverse temperature crystallization method and performed a comprehensive investigation of their phase transitions and dynamics of ferroelastic domain walls. Our results show that the crystals undergo reversible structural transitions from cubic (*Pm*$\overset{-}{3}$*m*) to tetragonal (*P*4/*mbm*), and further to orthorhombic (*Pnma*) on cooling (and the reverse on heating). In situ polarized optical microscopy clearly reveals that strip-like ferroelastic domain walls emerge in the room-temperature orthorhombic phase, become denser in the intermediate tetragonal phase, and vanish in the high-temperature cubic phase, with this evolution being reversible on thermal cycling. Dielectric measurements uncover frequency-dependent loss peaks at ∼170–180 K, which we attribute to the freezing of polarized ferroelastic domain walls pinned by chloride vacancies. Sharp anomalies in the dielectric permittivity at the structural transition temperatures are explained by the disappearance and reappearance of these domain walls near the structural transitions. Overall, our study clarifies the intrinsic phase behavior of CsPbCl_3_ and highlights the significant role of ferroelastic domain walls in its dielectric properties and relaxation. These insights provide a deeper understanding of CsPbCl_3_ and inform its potential optimization for blue-light emission [72] and other optoelectronic applications.

## Data Availability

The data presented in this study are available on request from the corresponding author.
